# Supplementary material for: Alteromonas Myovirus V22 Represents a New Genus of Marine Bacteriophages Requiring a Tail Fiber Chaperone for Host Recognition
Source: mSystems. 2020 Jun 9;5(3):e00217-20. doi: 10.1128/mSystems.00217-20 (PMC7289586; doi:10.1128/mSystems.00217-20)
Supplement: TABLE S1 [file mSystems.00217-20-st001.docx]

| **Strain** | **Genome**  **length (Mb)** | **GC content**  **(%)** | **No. of**  **CDS** | **No. of**  **tRNAs** | **No. of**  **rRNAs** | **Coding**  **DNA (%)** | **Total intergenic distance (kb)** |
| --- | --- | --- | --- | --- | --- | --- | --- |
| *A.* *mediterranea* PT11 | 4.34 | 44.84 | 3,677 | 64 | 4 | 88.00 | 514.36 |

**Table S1.** *Alteromonas mediterranea* PT11 genomic features.
