# Supplementary figures and images for: Alteromonas Myovirus V22 Represents a New Genus of Marine Bacteriophages Requiring a Tail Fiber Chaperone for Host Recognition
Source: mSystems. 2020 Jun 9;5(3):e00217-20. doi: 10.1128/mSystems.00217-20 (PMC7289586; doi:10.1128/mSystems.00217-20)

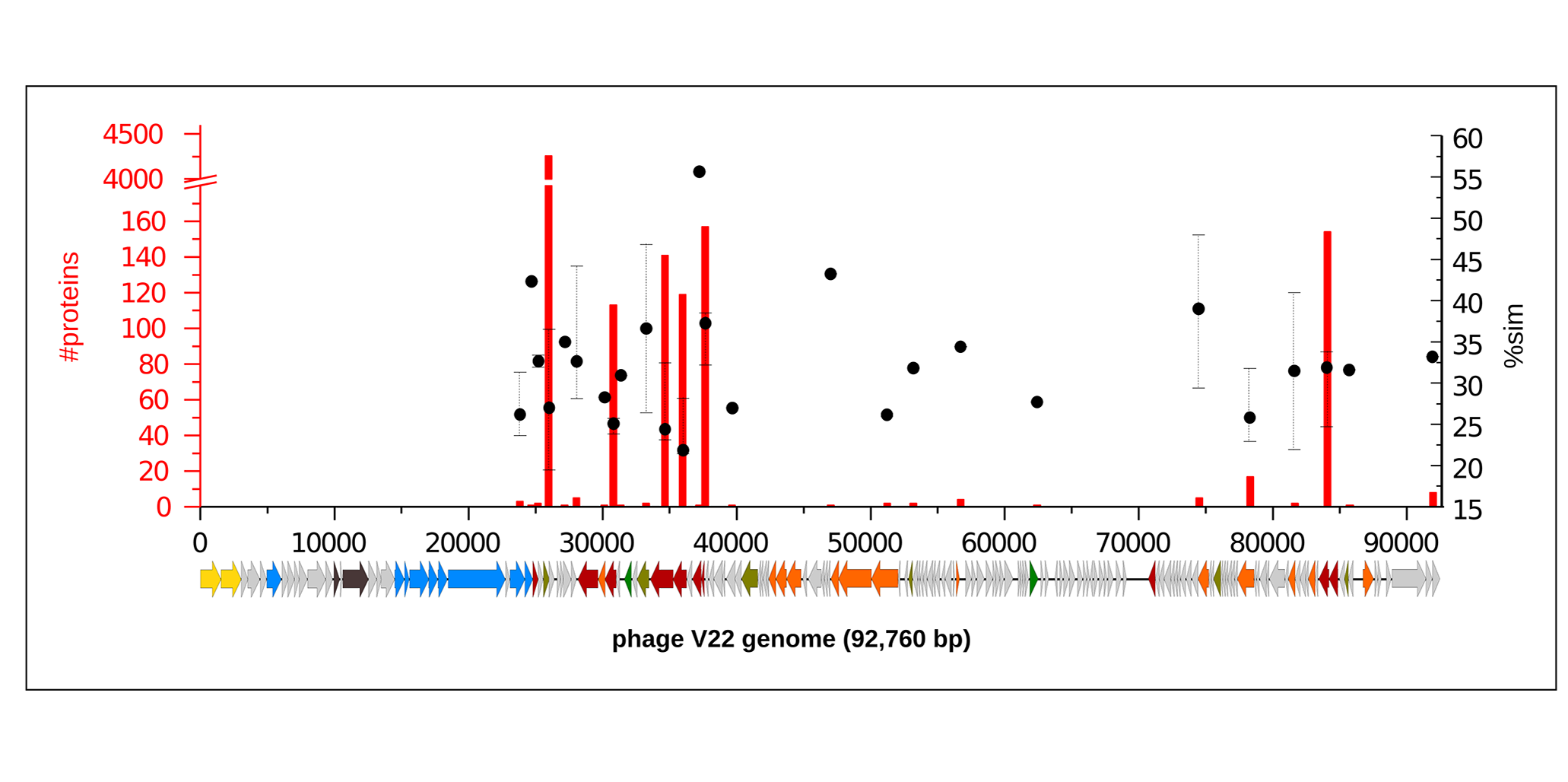

Supplement: FIG S1 [file mSystems.00217-20-sf001.tif]

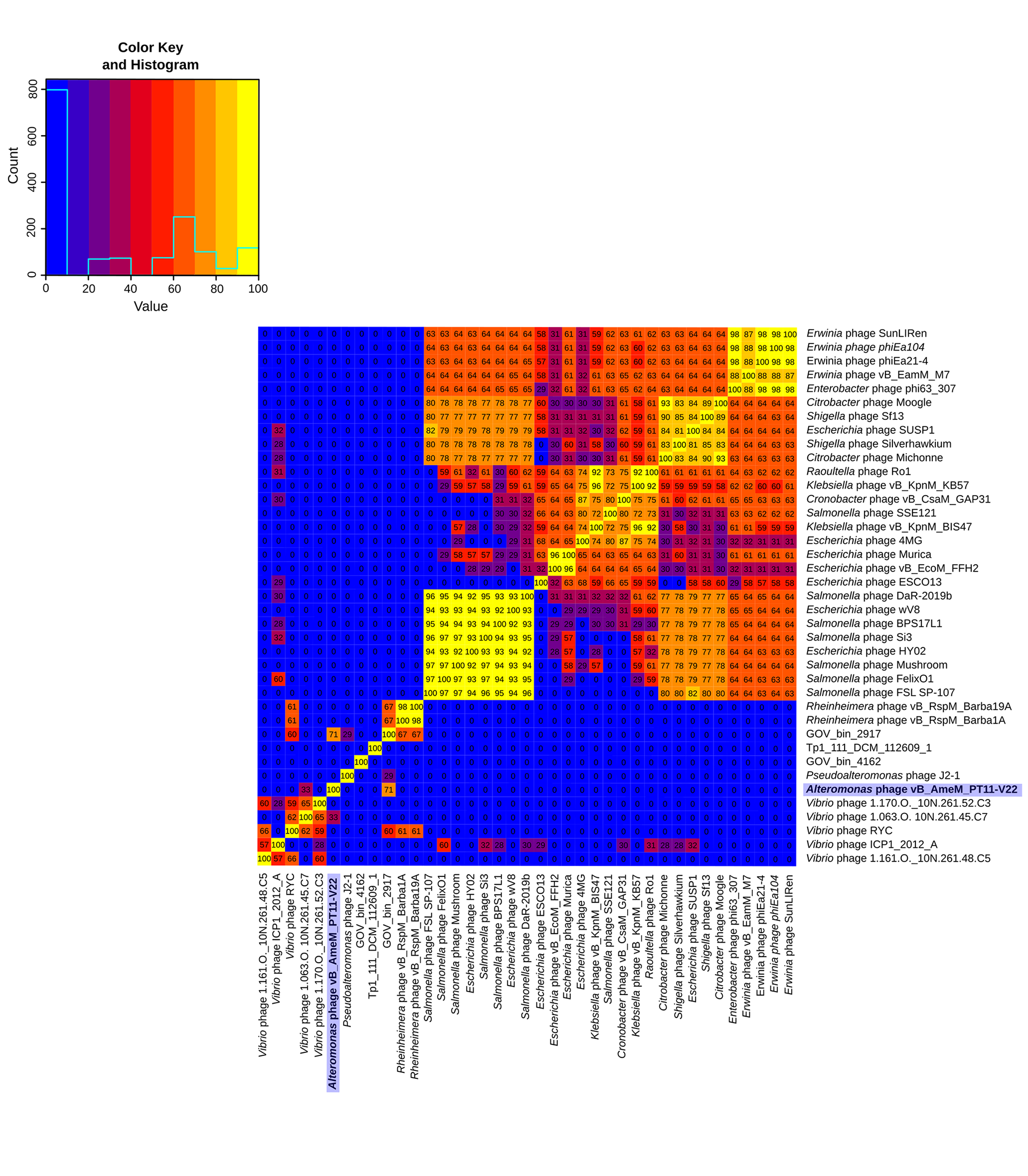

Supplement: FIG S2 [file mSystems.00217-20-sf002.tif]

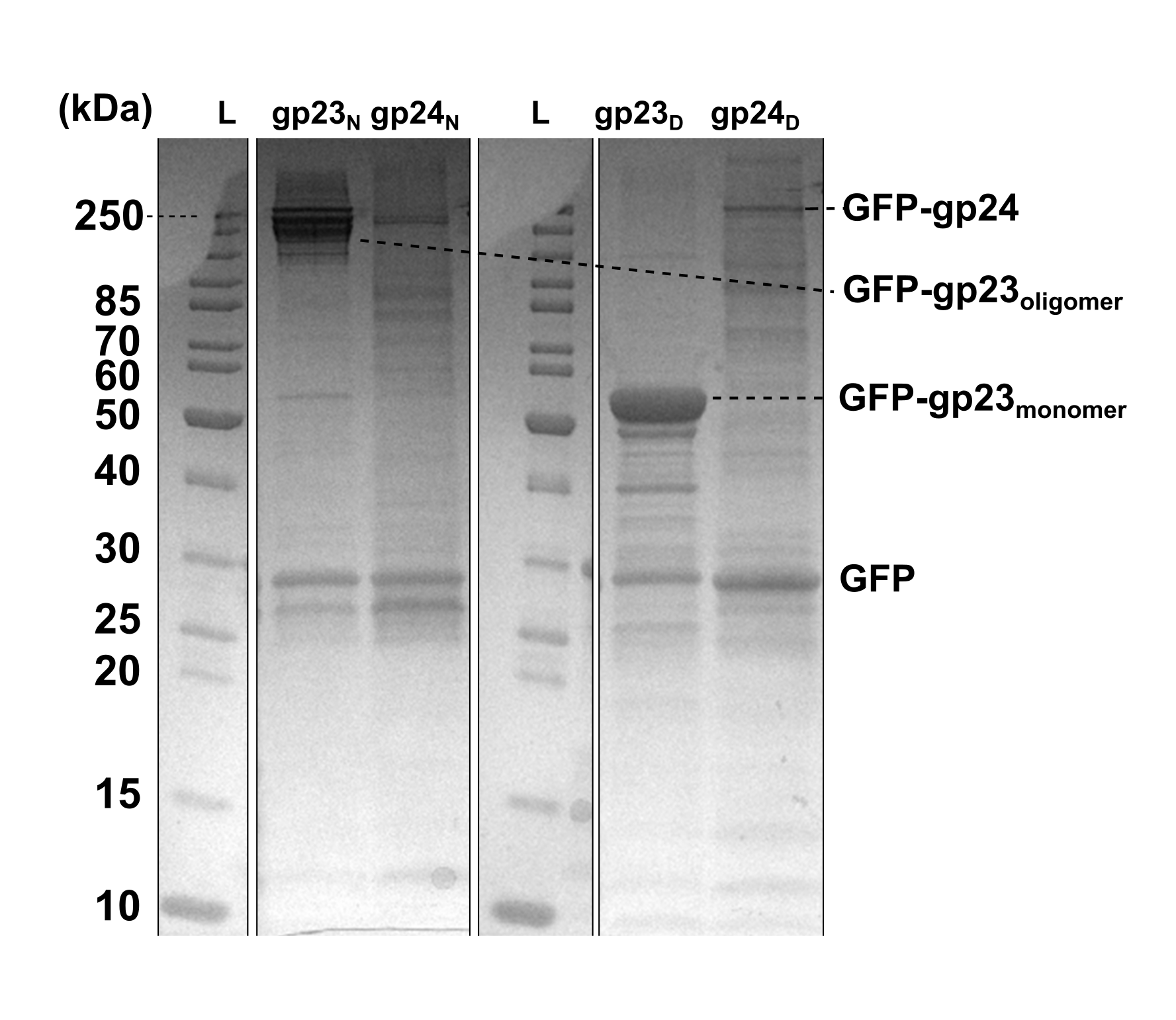

Supplement: FIG S3 [file mSystems.00217-20-sf003.tif]

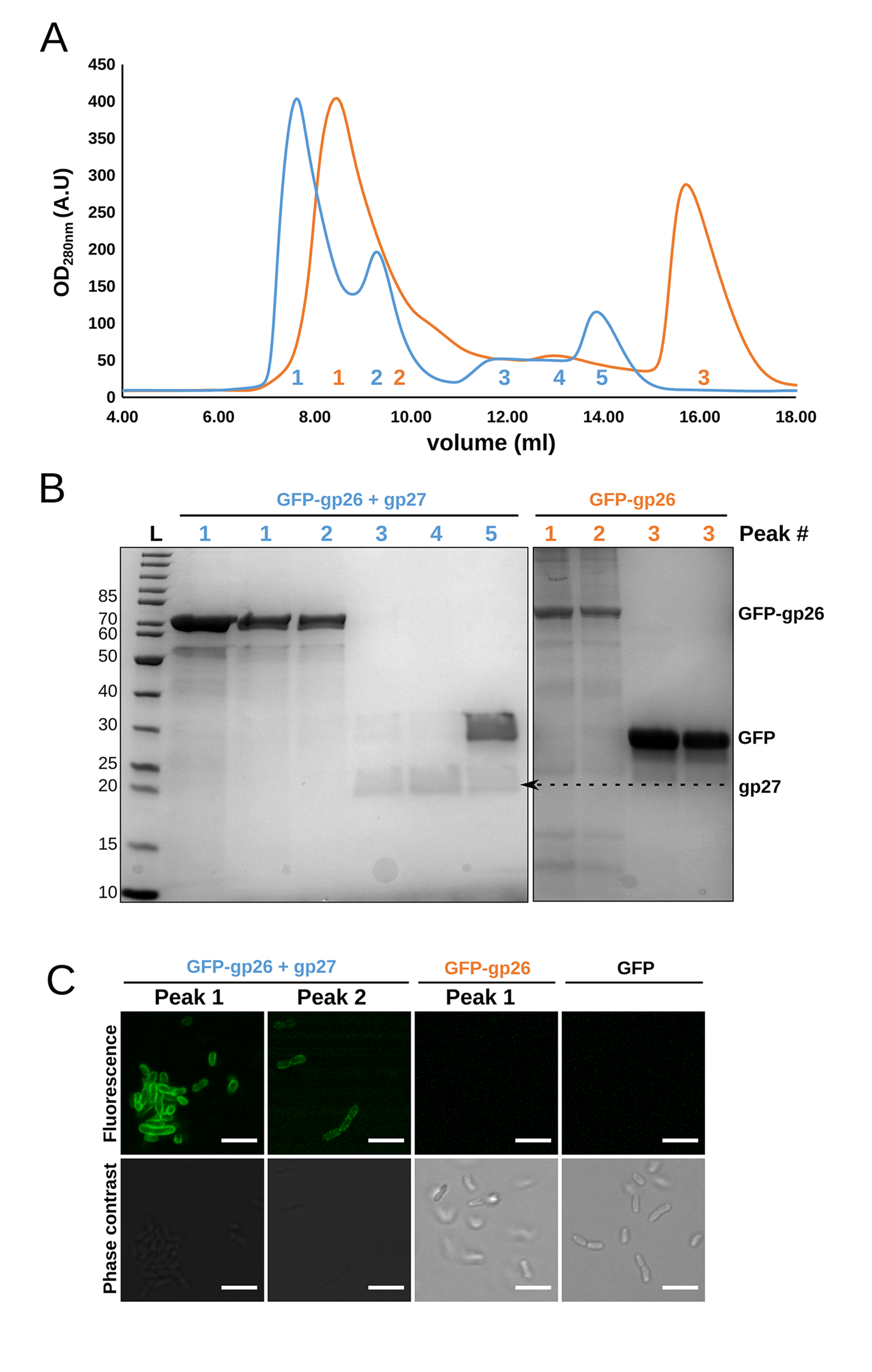

Supplement: FIG S4 [file mSystems.00217-20-sf004.tif]

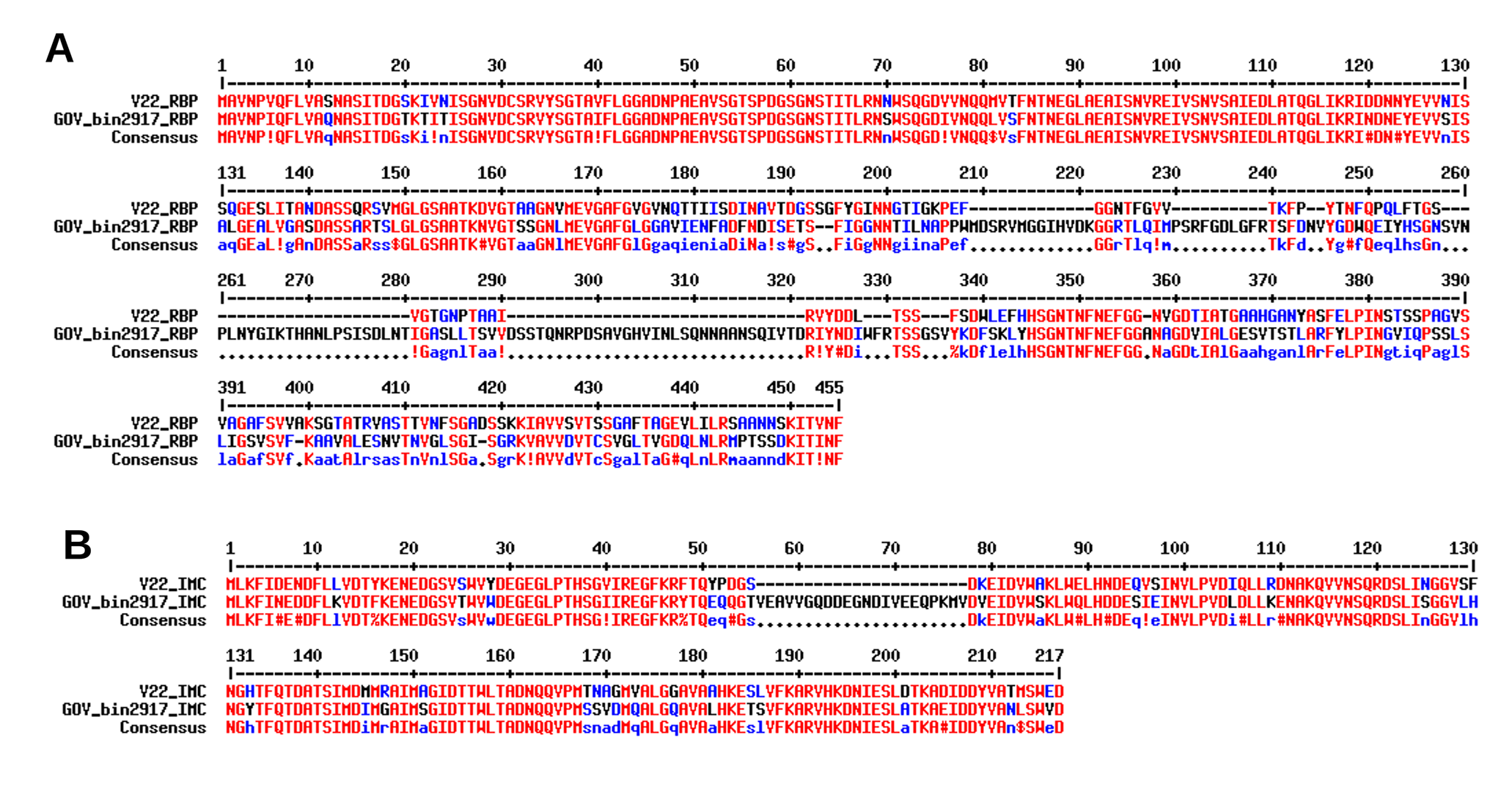

Supplement: FIG S5 [file mSystems.00217-20-sf005.tif]
