# Supplementary material for: Alteromonas Myovirus V22 Represents a New Genus of Marine Bacteriophages Requiring a Tail Fiber Chaperone for Host Recognition
Source: mSystems. 2020 Jun 9;5(3):e00217-20. doi: 10.1128/mSystems.00217-20 (PMC7289586; doi:10.1128/mSystems.00217-20)
Supplement: TABLE S2 [file mSystems.00217-20-st002.docx]

**Table S2.** V22 structural genes, AMGs and other metabolism-related genes annotated at non-redundant NCBI database (BLASTp ≥ 30%). Best hits (excluding GOV_bin_2917) with % similarity and % coverage are indicated.

| **V22 gene** | **Annotation** | **Organism *** | **% similarity** | **% query coverage** | **Accession**  **number** |
| --- | --- | --- | --- | --- | --- |
| CDS-1 | terminase | *Rheinheimera* phage vB_RspM_Barba19A | 62 | 85 | QCQ61943 |
| CDS-2 | portal | *Vibrio* phage qdvp001 | 60 | 95 | YP_009222143 |
| CDS-13 | tape measure chaperone | *Citrobacter* phage Michonne | 49 | 79 | YP_009612613 |
| CDS-15 | tape measure | *Pseudoalteromonas* phage J2-1 | 55 | 54 | ATN93398 |
| CDS-23 | baseplate | *Lactococcus* phage vB_Llc_bIBBF14 | 74 | 24 | ASR76011 |
| CDS-26 | tail fiber | *Enterobacter cloacae* | 52 | 27 | CZY36109 |
| CDS-28 | permuted papain-like amidase | *Cupriavidus plantarum* | 52 | 81 | REE92634 |
| CDS-30 | histidine kinase /response regulator | *Muricauda* sp. | 64 | 76 | RNC89645 |
| CDS-36 | nicotinate phosphoribosyl-transferase | *Vibrio* phage qdvp001 | 75 | 100 | YP_009222232 |
| CDS-38 | ribose phosphate pyrophosphokinase | *Rheinheimera* phage vB_RspM_Barba19A | 61 | 99 | QCQ61874 |
| CDS-40 | anti-sigma factor | *Pseudomonas* phage ventosus | 66 | 61 | ATW58321 |
| CDS-42 | Band 7 (stomatin-like) | *Endozoicomonas arenosclerae* | 70 | 96 | WP_062265680 |
| CDS-43 | ribonucleotide reductase (large subunit) | *Alteromonas* virus vB_AspP-H4/4 | 70 | 98 | ASL24402 |
| CDS-44 | ribonucleotide reductase (small subunit) | *Alteromonas* virus vB_AspP-H4/4 | 69 | 97 | ASL24401 |
| CDS-46 | thymidylate synthase | *Alteromonas* sp. | 74 | 94 | MAI39418 |
| CDS-47 | thioredoxin | *Pseoudomonas* phage O4 | 63 | 97 | YP_009304505 |
| CDS-54 | metallo-dependent phosphatase | *Rheinheimera* phage vB_RspM_Barba1A | 74 | 97 | QCQ57859 |
| CDS-72 | TM2 | *Notoacmeibacter marinus* | 61 | 80 | WP_114390415 |
| CDS-113 | 20S proteasome | *Rhodopseudomonas* palustris | 50 | 63 | WP_013502666 |
| CDS-126 | DprA | *Shewanella* phage 1/4 | 63 | 88 | YP_009100322 |
| CDS-144 | metallophosphoesterase | *Vibrio* phage 1.168.O._10N.261.52.A10 | 77 | 97 | AUR92013 |
| CDS-146 | PhoH | *Pseudomonas* phage VCM | 62 | 91 | YP_009222724 |
| CDS-149 | YopX | *Vibrio* phage 1.106.O._10N.286.51.F7 | 52 | 96 | AUR87964 |
