# Supplementary material for: Alteromonas Myovirus V22 Represents a New Genus of Marine Bacteriophages Requiring a Tail Fiber Chaperone for Host Recognition
Source: mSystems. 2020 Jun 9;5(3):e00217-20. doi: 10.1128/mSystems.00217-20 (PMC7289586; doi:10.1128/mSystems.00217-20)
Supplement: TABLE S3 [file mSystems.00217-20-st003.docx]

| **Host**  **(genus)** | **Phage** | **Size (bp)** | **Number of CDS** | **%GC** | **Subfamily**  **or genus** | **No. of**  **syntenic genes** | **Accession** |
| --- | --- | --- | --- | --- | --- | --- | --- |
| *Citrobacter* | Moogle | 87,999 | 131 | 38.98 | *Ounavirinae* | 21 | NC_027293 |
| *Citrobacter* | Michonne | 90,000 | 143 | 38.85 | *Ounavirinae* | 21 | NC_028247 |
| *Cronobacter* | vB_CsaM_GAP31 | 147,940 | 255 | 46.30 | *Vequintavirinae* | 18 | NC_019400 |
| *Enterobacter* | phi63_307 | 85,263 | 118 | 43.78 | *Ounavirinae* | 20 | MG589384 |
| *Erwinia* | vB_EamM_M7 | 84,694 | 115 | 43.39 | *Ounavirinae* | 20 | NC_041978 |
| *Erwinia* | SunLIRen | 84,559 | 115 | 43.75 | *Ounavirinae* | 20 | MH426725 |
| *Erwinia* | phiEa104 | 84,565 | 116 | 43.83 | *Ounavirinae* | 19 | NC_015292 |
| *Erwinia* | phiEa21-4 | 84,576 | 116 | 43.81 | *Ounavirinae* | 19 | NC_011811 |
| *Erwinia* | vB_EamM_Y2 | 56,621 | 92 | 44.23 | unclassified | 10 | NC_019504 |
| *Escherichia* | HY02 | 86,252 | 121 | 38.92 | *Ounavirinae* | 21 | NC_028872 |
| *Escherichia* | wV8 | 88,487 | 127 | 38.89 | *Ounavirinae* | 20 | NC_012749 |
| *Escherichia* | SUSP1 | 90,743 | 137 | 39.76 | *Ounavirinae* | 21 | NC_028808 |
| *Escherichia* | vB_EcoM_FFH2 | 139,020 | 216 | 43.61 | *Vequintavirinae* | 18 | NC_024134 |
| *Escherichia* | Murica | 135,391 | 206 | 43.61 | *Vequintavirinae* | 13 | NC_041871 |
| *Escherichia* | 4MG | 148,567 | 255 | 46.33 | *Vequintavirinae* | 18 | NC_022968 |
| *Escherichia* | ESCO13 | 149,813 | 278 | 39.13 | unclassified | 17 | KX552041 |
| *Klebsiella* | vB_KpnM_BIS47 | 147,443 | 260 | 44.62 | *Vequintavirinae* | 19 | KY652726 |
| *Klebsiella* | vB_KpnM_KB57 | 142,987 | 245 | 44.62 | *Vequintavirinae* | 18 | NC_028659 |
| *Pseudoalteromonas* | J2-1 | 142,204 | 183 | 37.92 | unclassified | 21 | MF988720 |
| *Raoultella* | Ro1 | 145,759 | 248 | 44.55 | *Vequintavirinae* | 17 | MG250486 |
| *Rheinheimera* | vB_RspM_Barba1A | 80,240 | 129 | 38.20 | unclassified | 16 | MK719701 |
| *Rheinheimera* | vB_RspM_Barba19A | 84,608 | 144 | 38.25 | unclassified | 16 | MK719730 |
| *Salmonella* | Si3 | 84,419 | 119 | 39.01 | *Ounavirinae* | 21 | NC_041922 |
| *Salmonella* | FSL SP-107 | 37,993 | 52 | 38.99 | *Ounavirinae* | 21 | KC139640 |
| *Salmonella* | DaR-2019b | 88,966 | 124 | 39.11 | *Ounavirinae* | 21 | MK965970 |
| *Salmonella* | Felix 01 | 86,155 | 121 | 39.01 | *Ounavirinae* | 21 | NC_005282 |
| *Salmonella* | Mushroom | 87,709 | 124 | 39.03 | *Ounavirinae* | 21 | NC_041923 |
| *Salmonella* | BPS17L1 | 84,916 | 120 | 38.86 | *Ounavirinae* | 21 | NC_042096 |
| *Salmonella* | SSE-121 | 147,745 | 239 | 45.29 | *Vequintavirinae* | 18 | NC_027351 |
| *Shigella* | Silverhawkium | 88,821 | 135 | 39.07 | *Ounavirinae* | 21 | MK562505 |
| *Shigella* | Sf13 | 87,570 | 135 | 38.91 | *Ounavirinae* | 20 | NC_042017 |
| *unknown* | GOV_bin-2917 | 93,482 | 161 | 36.07 | *Alterovirus* | 46 | MK892806 |
| unknown | GOV_bin-4162 | 86,285 | 144 | 39.69 | unclassified | 22 | MK892724 |
| unknown | Tp1_111_DCM_112609_1 | 87,101 | 140 | 43.24 | unclassified | 12 | MK892832 |
| *Vibrio* | RYC | 158,066 | 262 | 39.04 | unclassified | 15 | AP014858 |
| *Vibrio* | 1.063.O. 10N.261.45.C7 | 128,641 | 231 | 37.34 | unclassified | 10 | MG592441 |
| *Vibrio* | 1.161.O.10N.261.48.C5 | 140,668 | 228 | 37.68 | unclassified | 8 | MG592529 |
| *Vibrio* | 1.170.O._10N.261.52.C3 | 133,692 | 214 | 38.70 | unclassified | 8 | MG592537 |
| *Vibrio* | ICP1_2012_A | 121,418 | 214 | 37.17 | unclassified | 16 | MH310936 |

**Table S3.** Main features of phages syntenic to V22. Some features of the 39 phage genomes syntenic to phage V22 identified in this study. GenBank accession numbers are indicated on the right.
