# Supplementary material for: Alteromonas Myovirus V22 Represents a New Genus of Marine Bacteriophages Requiring a Tail Fiber Chaperone for Host Recognition
Source: mSystems. 2020 Jun 9;5(3):e00217-20. doi: 10.1128/mSystems.00217-20 (PMC7289586; doi:10.1128/mSystems.00217-20)
Supplement: TABLE S4 [file mSystems.00217-20-st004.docx]

**Table S4.** Analyzed genomes non-syntenic to V22.

| **Host**  **(genus)** | **Phage** | **Family** | **Subfamily or genus** | **Accession** |
| --- | --- | --- | --- | --- |
| *Escherichia* | vB_EcoM-FV3 | *Myoviridae* | *Vequintavirinae* | NC_019517 |
| *Escherichia* | Mu | *Myoviridae* | *Muvirus* | NC_000929 |
| *Escherichia* | P2 | *Myoviridae* | *Peduovirinae* | NC_041848 |
| *Escherichia* | vB_EcoM_Schickermooser | *Myoviridae* | unclassified | MK373788 |
| *Escherichia* | vB_EcoM-Ro121c4YLVW | *Myoviridae* | unclassified | MH051333 |
| *Escherichia* | vB_EcoM-ECP32 | *Myoviridae* | *Vequintavirinae* | MK883718 |
| *Escherichia* | rv5 | *Myoviridae* | *Vequintavirinae* | NC_011041 |
| *Escherichia* | phAPEC8 | *Myoviridae* | unclassified | NC_020079 |
| *Escherichia* | T4 | *Myoviridae* | *Tevenvirinae* | NC_000866 |
| *Pelagibacter* | HTVC008M | *Myoviridae* | unclassified | YP_007517946 |
| *Prochlorococcus* | P-HM1 | *Myoviridae* | unclassified | NC_015280 |
| *Prochlorococcus* | Syn1 | *Myoviridae* | unclassified | NC_015288 |
| *Prochlorococcus* | P-SSM4 | *Myoviridae* | unclassified | NC_006884 |
| *Proteus* | Mydo | *Myoviridae* | *Vequintavirinae* | MK024806 |
| *Pseudoalteromonas* | PH357 | *Myoviridae* | unclassified | KX822733 |
| *Rheinheimera* | vB_RspM_Barba31A | *Myoviridae* | unclassified | MK719750 |
| *Salmonella* | 19 | *Myoviridae* | unclassified | NC_029072 |
| *Sinorhizobium* | phiM12 | *Myoviridae* | *Emdodecavirus* | NC_027204 |
| *Synechococcus* | syn9 | *Myoviridae* | unclassified | NC_008296 |
| *Synechococcus* | S-SM2 | *Myoviridae* | unclassified | NC_015279 |
| *Vibrio* | ICP1_2001_A | *Myoviridae* | unclassified | HQ641353 |
| *Vibrio* | S4-7 | *Myoviridae* | unclassified | KX507046 |
| *Vibrio* | 1.084.O._10N.261.49.F5 | *Myoviridae* | unclassified | MG592459 |
| *Vibrio* | ICP1_2006_E | *Myoviridae* | unclassified | MH310934 |
| *Vibrio* | pVp-1 | *Siphoviridae* | *Cetovirus* | NC_019529 |
| *Vibrio* | Ceto | *Siphoviridae* | *Cetovirus* | NC_042094 |
| *Vibrio* | vB_VorS-PVo5 | *Siphoviridae* | *Cetovirus* | KT345706 |
