# Supplementary material for: Alteromonas Myovirus V22 Represents a New Genus of Marine Bacteriophages Requiring a Tail Fiber Chaperone for Host Recognition
Source: mSystems. 2020 Jun 9;5(3):e00217-20. doi: 10.1128/mSystems.00217-20 (PMC7289586; doi:10.1128/mSystems.00217-20)
Supplement: TABLE S5 [file mSystems.00217-20-st005.docx]

**Table S5.** Oligonucleotides used to generate the protein expression plasmids.

| **Construct** | **Forward primer (5´- 3´)** | **Reverse primer (5´- 3´)** |
| --- | --- | --- |
| *pQE30_HGT* backbone | GCCCTGGAAATACAGATTCTCG | AATTAGCTGAGCTTGGACTCCT |
| *gp23* insert | CGAGAATCTGTATTTCCAGGGCATGGCAATTAATGCAGATAAC | AGGAGTCCAAGCTCAGCTAATTTTACGCGGTTCTTACCCATA |
| *gp24* insert | CGAGAATCTGTATTTCCAGGGCATGGCACAATTTGAGATTACA | AGGAGTCCAAGCTCAGCTAATTCTATGCAAGTTTGTTTTCTTCG |
| *gp26* insert | CGAGAATCTGTATTTCCAGGGCATGGCTGTAAATCCAGTACA | AGGAGTCCAAGCTCAGCTAATTTTAGAAGTTTACTGTTATTTTAGAGTTGT |
| *gp26_gp27* insert | CGAGAATCTGTATTTCCAGGGCATGGCTGTAAATCCAGTACA | AGGAGTCCAAGCTCAGCTAATTTTAGTCCTCCCAAGACATTG |
